# Efficient Rejoining of DNA Double-Strand Breaks despite Increased Cell-Killing Effectiveness following Spread-Out Bragg Peak Carbon-Ion Irradiation

**DOI:** 10.3389/fonc.2016.00028

**Published:** 2016-02-12

**Authors:** Nicole B. Averbeck, Jana Topsch, Michael Scholz, Wilma Kraft-Weyrather, Marco Durante, Gisela Taucher-Scholz

**Affiliations:** ^1^Department of Biophysics, GSI Helmholtzzentrum für Schwerionenforschung GmbH, Darmstadt, Germany; ^2^Technische Universität Darmstadt, Darmstadt, Germany

**Keywords:** heavy ions, carbon-ion radiotherapy, DSB complexity, DSB repair, error-prone DNA repair, RBE

## Abstract

Radiotherapy of solid tumors with charged particles holds several advantages in comparison to photon therapy; among them conformal dose distribution in the tumor, improved sparing of tumor-surrounding healthy tissue, and an increased relative biological effectiveness (RBE) in the tumor target volume in the case of ions heavier than protons. A crucial factor of the biological effects is DNA damage, of which DNA double-strand breaks (DSBs) are the most deleterious. The reparability of these lesions determines the cell survival after irradiation and thus the RBE. Interestingly, using phosphorylated H2AX as a DSB marker, our data in human fibroblasts revealed that after therapy-relevant spread-out Bragg peak irradiation with carbon ions DSBs are very efficiently rejoined, despite an increased RBE for cell survival. This suggests that misrepair plays an important role in the increased RBE of heavy-ion radiation. Possible sources of erroneous repair will be discussed.

## Introduction

Radiotherapy is an indispensable tool for treating solid tumors (1). Advances in conventional radiation therapy with photons and especially new approaches using charged particles have led to an improved physical delivery of dose in radiation therapy (2–4). Irradiation with accelerated ions heavier than protons, namely carbon ions, has additional advantage as it is characterized by an increased relative biological effectiveness (RBE) in the targeted tumor volume (4). This allows the irradiation of deep-seated tumors, minimizing at the same time the dose to normal tissue or in organs at risk (2). Accelerated ions of a linear energy transfer (LET) of >10 keV/μm are considered high-LET radiation. Due to their characteristic energy deposition within a confined volume, they cause DNA damage of greater complexity (5–7). A special feature of this densely ionizing radiation is the induction of clustered lesions – two or more DNA lesions within one or two helix turns (8) – comprising double-strand breaks (DSBs) in close proximity that are more difficult to repair (9, 10). An additional level of complexity arises due to the localized microscopic energy deposition occurring along the particle track when traversing nuclear chromatin. At different size scales, from the nucleosome to chromatin fiber loops, the induction of spatially correlated DSBs within chromatin subunits can increase the severity of the induced lesions (11, 12), resulting in a decreased probability of DSB repair (13). Damage clustering at different levels is thus a crucial factor for the enhanced biological effects of radiotherapeutical heavy-ion irradiation and was shown earlier (14, 15).

Several studies have analyzed the repair capacity of heavy ion radiation-induced DSBs with different kinds of methods (13, 16–20). All studies revealed that with increasing LET repair slows down and the number of DSBs remaining unrepaired increases. Furthermore, chromosome studies applying premature chromosome condensation (PCC) on cells exposed to radiation of different LET agree with these data; with increasing LET, the fraction of excess PCC fragments increases and correspondingly the unrejoined breaks (21–25). In addition, high-LET radiation is also more effective in inducing mutations and chromosome aberrations, especially of the complex type, i.e., involving at least two or more chromosomes, which indicates misrepair of DSBs (26–30). Likely sources for misrepair are the close proximity of the breaks, which could facilitate the ligation of wrong break ends and the choice of the DSB-repair pathway (7, 31). The latter is supported by our findings that repair of carbon ion-induced DSBs is dependent on resection (32), a process that clearly influences the repair pathway choice (33). Thus, the increased RBE of high-LET radiation is presumably based on an increased number of unrejoined and misrepaired DSBs.

In carbon-ion radiotherapy the target volume is typically irradiated with ions from opposing fields. Beams with different ion energies are superimposed, resulting in a spread-out Bragg peak (SOBP) with the desired homogeneous distribution of dose (4). Consequently, the cells within the SOBP are exposed to a wide spectrum of carbon ions with different individual energies and LET. Due to this mixed radiation field, DNA damage of different complexity is expected to occur, from rather simple lesions induced by high-energy ions to very complex damage induced by low-energy ions. The DNA damage of different quality will likely influence the efficiency of cell killing and thus the RBE.

Earlier survival studies have shown that the RBE depends on the capacity to repair the induced DNA damage (14, 15). These and most of the above mentioned research, which revealed an increased number of unrejoined DSBs in repair studies and misrepaired DSBs in cytogenetic analyses, was performed using mainly monoenergetic ions or hamster cells (13, 16–20, 26–29). Aimed at a better understanding of the relationship between DSB repair and the RBE of therapeutic carbon-ion irradiation, we examined the effect of radiation quality on the survival of human fibroblasts and DSB repair.

## Results

Within this study, we used normal human fibroblasts to first examine the systematics of survival depending on the changing radiation quality along the penetration path of carbon ions. Furthermore, we compared the repair of DSBs after exposure to the different radiation qualities in the carbon-ion entrance channel (EC) and SOBP, where the target tumor volume would be seated. The confluent fibroblast cells analyzed in this study preclude the interference of cell cycle changes and are thus especially suitable for reliable repair measurements using phosphorylated H2AX (γH2AX) as a marker for DSBs (34).

### Cell Survival in Dependence of the Penetration Depth of Carbon Ions

**Figure 1:**
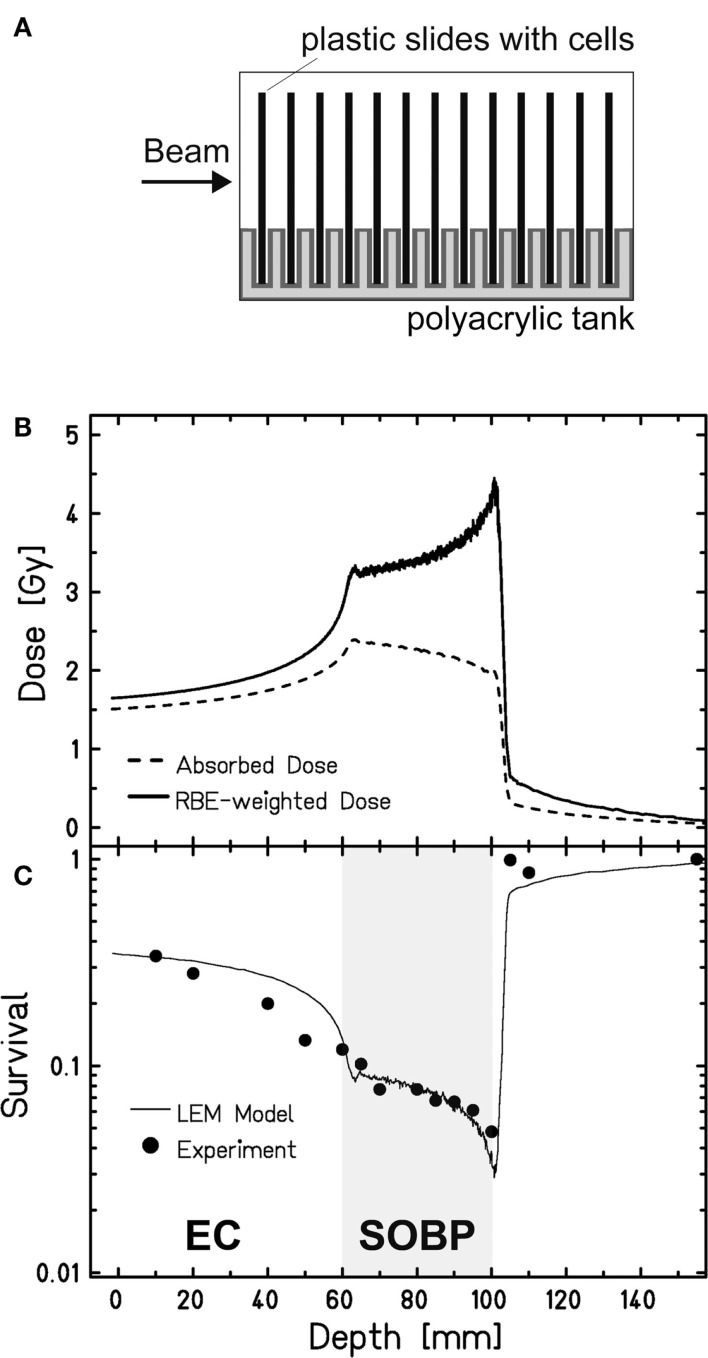
**Survival of human fibroblasts upon a one-field carbon-ion irradiation along the beam penetration path**. **(A)** Experimental setup for a one-field irradiation. Confluent, human fibroblasts (AG1522D) grown on slides were placed perpendicular to the beam direction in a medium filled tank at different positions from the beam entrance side, as described earlier (35). The irradiation at the heavy-ion synchrotron (SIS), GSI Darmstadt, was done with a 4-cm SOBP in a water-equivalent depth of 6–10 cm (LET: 45 keV/μm at the proximal edge, 150 keV/μm at the distal edge). **(B)** Depth-dose distribution of the absorbed dose (dashed line) and the RBE-weighted dose (LEM calculated; solid line) of the carbon ions. **(C)** Measured (circles; *n* = 1) and calculated (line; LEM) clonogenic cell survival of human fibroblasts along the beam axes.

To study cell survival along the carbon-ion EC and SOBP, we applied an experimental setup that allows irradiating cells at different positions within a polyacrylic tank previously described (35) (Figure 1A). Following the one-field irradiation with a 4-cm SOBP of carbon ions in a water-equivalent depth of 6–10 cm, the survival data obtained for confluent, human fibroblasts show the expected depth profile with higher survival levels in the EC and a decline of cell survival in the target SOBP region, yielding a region with clearly reduced cell survival compared to the EC (Figure 1C). The increase of the RBE with penetration depth – represented by the ratio of the two depth-dose curves in Figure 1B – becomes obvious from the fact that despite the reduction of absorbed dose toward the distal end of the SOBP the biological effect still increases, i.e., the survival drops within the SOBP. The RBE reaches a value of 2.3 at the distal edge, whereas in the EC, it is approximately 1.1.

These data have been also used to validate the local effect model (LEM) that has been developed for biological optimization in treatment planning (35). Very good agreement is found between the model prediction and the experimental data both in the EC and in the target region.

### Repair Kinetics of DSBs Induced in the Carbon-Ion EC and SOBP

**Figure 2:**
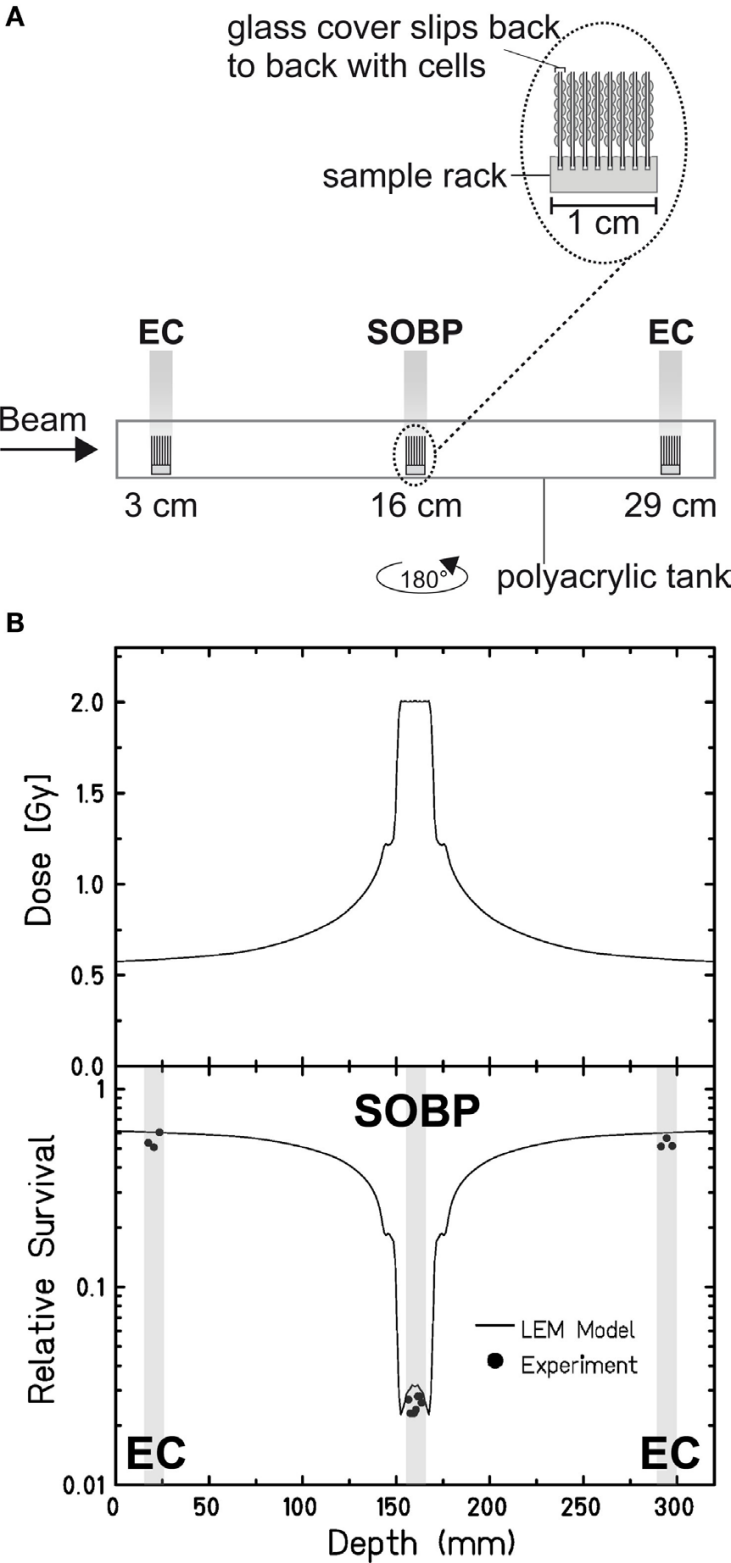
**Verification of the experimental setup to mimic therapy-like carbon-ion irradiation**. **(A)** Experimental setup for a two-field irradiation. Confluent, human fibroblasts (AG1522D) were exposed at different positions from the beam entrance side within a medium filled tank. At the SIS, GSI Darmstadt, the two-field configuration typical for patient irradiation was simulated by irradiating the tank from both sides with a horizontal turn of 180°. The irradiation was done with an SOBP of 2.4 cm at a water-equivalent depth of 16 cm. The dose in the SOBP was 2 Gy (dose-averaged LET: 70–85 keV/μm). Samples in the EC region were irradiated with a corresponding dose of 0.6 Gy (dose-averaged LET: 13 keV/μm). **(B)** Top: distribution of the absorbed dose upon two-sided therapy-like irradiation. Bottom: corresponding calculated (line; LEM) and measured (circles; *n* = 1) cell survival. The gray boxes indicate the position of the samples during irradiation.

**Figure 3:**
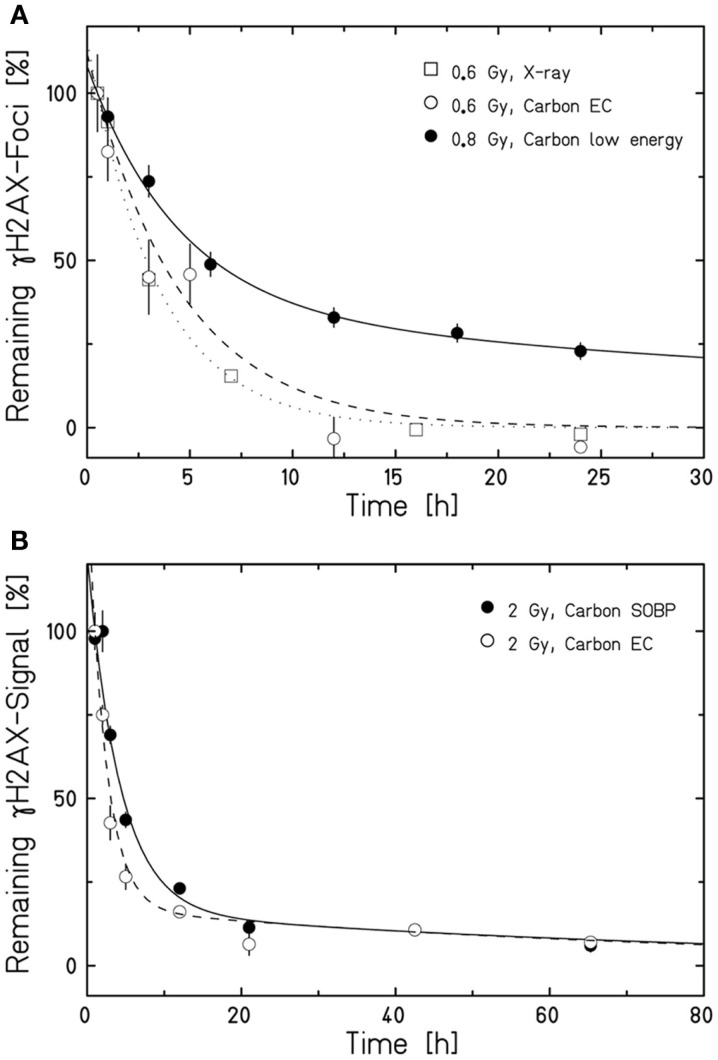
**Human fibroblasts repair DSBs induced by therapy-like carbon-ion irradiation**. DSB-repair kinetics of confluent (G0/G1-phase) human AG1522 fibroblasts was measured after exposure to different radiation qualities. The average γH2AX-foci number **(A)** or γH2AX signal **(B)** of mock irradiated cells was subtracted from all data measured after irradiation. The curves are a guide to the eye obtained by exponential fits after normalization to the initial or extrapolated γH2AX values at 30 min **(A)** or 1 h **(B)** post irradiation. Data points represent the average of 2–4 experiments ± SEM [exception: low-energy carbon-ion irradiation in **(A)**; *n* = 1 ± SEM foci number/nucleus, at least 100 cells were analyzed]. **(A)** Kinetics after irradiation with 0.6 Gy X-rays, 0.6 Gy carbon ions in the EC (for irradiation conditions see Figure 2A), or 0.8 Gy low-energy carbon ions almost parallel to the cell monolayer (9.9 MeV/u on target; 168 keV/μm). DSBs were revealed by a γH2AX-foci analysis after γH2AX immunostaining performed as described in Meyer et al. (42). **(B)** Comparison of the DSB-repair capacity in human fibroblasts after 2 Gy carbon-ion irradiation in the EC or SOBP (for irradiation conditions see Figure 2A). The global immunofluorescent γH2AX signal was analyzed by flow cytometry according to Tommasino et al. (43).

Aimed at mimicking a therapy-like configuration, we studied the DSB-repair capacity of confluent (G0/G1-phase) human fibroblasts upon a two-field SOBP carbon-ion irradiation. The irradiation from two opposing sides, typical for patient treatment, has the advantage of compensating for the variations in LET and RBE gradients observed in Figure 1. The applied physical dose within the SOBP was 2 Gy according to a typical therapeutic fraction; the corresponding EC dose was 0.6 Gy. We adapted the previously described experimental setup (Figure 1A) placing cells grown on coverslips (to allow DSB microscopy analysis; see below) at positions equivalent to those in the EC and the SOBP (Figure 2A). The irradiation geometry was verified by the measured clonogenic cell survival. The experimental data showing clearly lower survival in the SOBP compared to the EC (Figure 2B, circles) agree very well with the calculated survival from the LEM (Figure 2B, line). In this case, it has been taken into account that cells growing on glass typically show a higher sensitivity as compared to cells grown on plastic material (36). Subsequently, this setup was used to measure the repair of DSBs induced in the EC and SOBP. We first directly compared the repair of DSBs induced by 0.6 Gy carbon ions in the EC with that after the same dose of X-rays (Figure 3A) using immunofluorescence microscopy to detect the DSB marker γH2AX (34). This method had proven most appropriate at the dose applied here and represents a suitable DSB-repair assay in G0/G1-phase cells (34). The repair of DSBs for both types of irradiation was similar and mostly completed within 12 h, as determined by γH2AX-foci loss. This agrees well with earlier findings on DSB rejoining along the irradiation axis of therapy-relevant carbon ions or X-rays (16). By contrast, following irradiation with a comparable dose (0.8 Gy) of high LET (168 keV/μm), low-energy (9.9 MeV/u on target) carbon ions, which correspond to stopping ions in the SOBP, a significant fraction of γH2AX foci is still remaining 24 h post exposure (Figure 3A). These results emphasize the impact of radiation quality on DSB repair and show that repair of clustered DSBs is impaired, which is in line with earlier findings (18–20, 37). It should be noted, however, that despite irradiation with the beam almost parallel to the cell monolayer enabling improved foci counting along the ion tracks, γH2AX foci induced by these densely ionizing ions may not represent individual DSBs (38–41).

We next applied the two-field carbon-ion irradiation to measure the repair of DSBs induced in the center of an SOBP at a dose of 2 Gy. In order to avoid inconsistencies in foci counting due to overlapping foci at the higher dose and LET (41, 44, 45), flow cytometry was used to quantify the global γH2AX signal. This method is suggested to give an enhanced resolution in measuring DSB damage induced by high-LET high-energy ion irradiation compared to γH2AX-foci counting (20, 46). The γH2AX signal was measured up to 65 h post exposure and the DSB-repair data for irradiation in the SOBP are compared with the corresponding γH2AX values obtained after exposure of the cells to the same dose (2 Gy) of ions in the EC (Figure 3B). As expected, DSBs induced within the EC are mostly repaired within 12 h, similar to the result obtained by the γH2AX-foci assay upon irradiation with 0.6 Gy. Interestingly, also the cells placed in the SOBP region repaired the carbon ion-induced DSBs very efficiently to the same extent as in the EC. Although the decay of the γH2AX signal appeared to be slightly slower up to 24 h post SOBP irradiation, it declined similar to the EC almost to control values within 48 h.

## Discussion

Here, we aimed at clarifying the relationship between DSB repair and RBE of therapeutic carbon-ion irradiation. We confirmed that the repair capacity represents an important factor in this relationship, and our data further suggest that the quality of the repair also affects the RBE.

### Efficiency of Cell Killing and DSB Rejoining along the Penetration Path of Carbon Ions

The survival data of the G0/G1-phase human fibroblasts and the calculated RBE in the EC and the SOBP demonstrate that the RBE is highest in the SOBP. This is in accordance with data obtained with hamster cells in a similar setup (35). Interestingly, the smallest survival and highest RBE are observed within the SOBP where the ion energy is smallest, at the very distal edge. Our repair data on ion irradiation of this quality, i.e., high LET, low-energy carbon ions (9.9 keV/u on target) in Figure 3A, show impaired repair of DSBs. Thus, the decreased survival at the distal edge of a one-field SOBP irradiation corresponds well with the decreased repair capacity we observed for low-energy ions and as it was seen earlier (16, 19). The notion that the decreased DSB-repair capacity is responsible for the decreased survival upon low-energy carbon-ion irradiation is further supported by earlier data on survival of confluent, human fibroblasts upon fractionated and non-fractionated irradiation with low-energy carbon ions (11 MeV/u, 153.5 keV/μm); fractionating the dose with a 24 h interval between fractions did not improve the survival indicating that the capacity to repair the induced DNA damage is very low (14).

The survival data and repair kinetics of cells irradiated within the EC (Figures 2 and 3) show that the cells can cope well with this irradiation. DSB rejoining is complete and its kinetics comparable to the rejoining kinetics of X-ray-induced DSBs (Figure 3A). This suggests that the repair is successful and hence ensures survival. This conclusion is supported by earlier work with the same cell system. This work revealed an RBE_10_ of 1.2 ± 0.3 for high-energy carbon ions (266 keV/u; 13.7 keV/μm) (14), which are in the range of carbon ions within the EC in the here presented experiment. In addition, Wang et al. showed clearly increased survival for both radiation qualities upon fractionated irradiation, which further corroborates that DNA damage in the EC can be effectively repaired (14).

**Figure 4:**
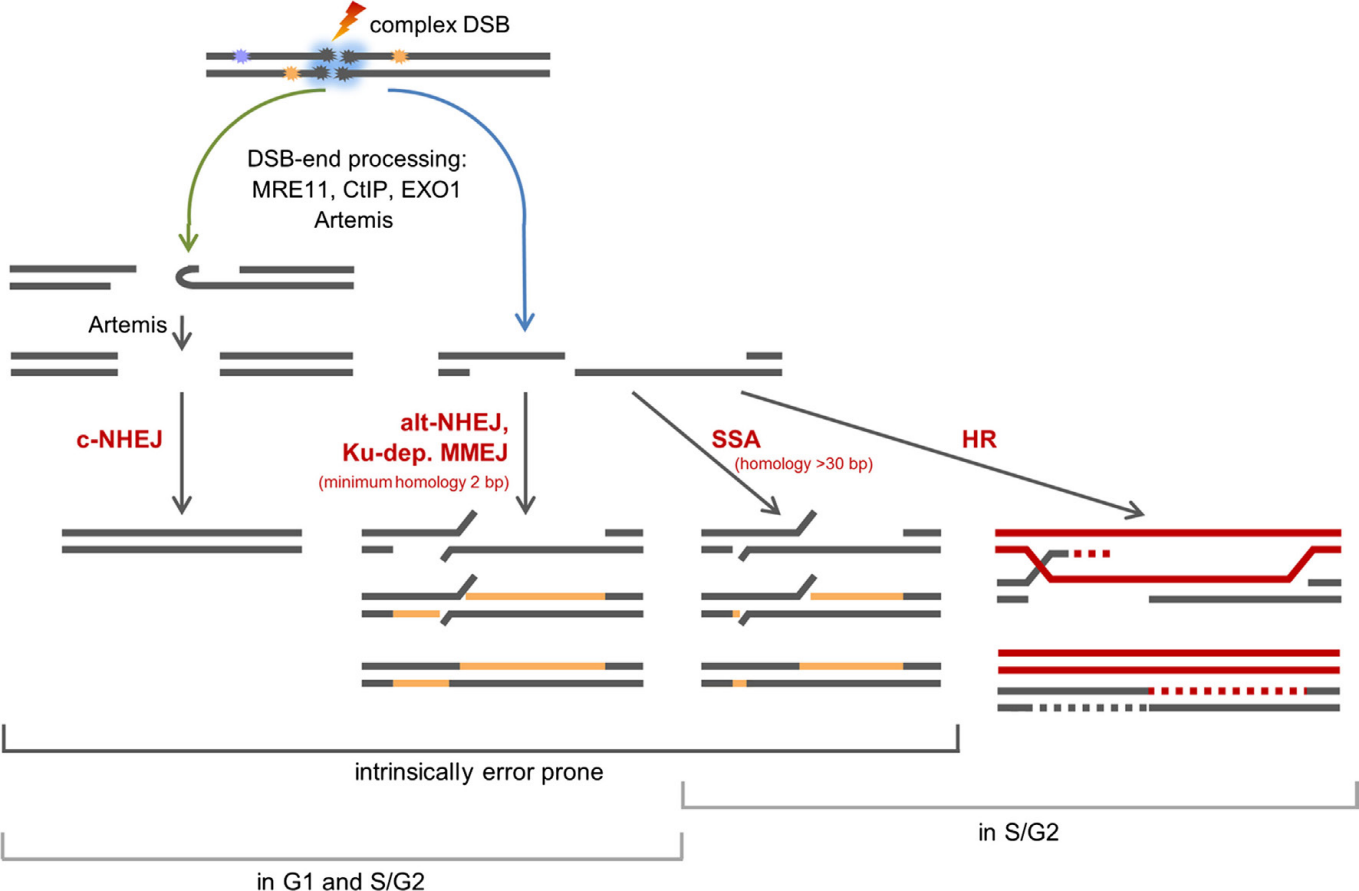
**Scheme of the repair of complex, carbon ion-induced DSBs**. DSBs that cannot be rapidly repaired by c-NHEJ will undergo break-end resection by MRE11, CtIP, EXO1, and break-end processing by Artemis. Blue arrow: resected DSBs can be repaired by alt-NHEJ, a potentially Ku-dependent MMEJ, SSA, or HR. The latter two pathways operate in S- and G2-phase only. Green arrow: Artemis may make resected DSB ends available for c-NHEJ.

Our data in Figure 2 revealed that the survival within the SOBP is smallest, yet DSBs induced within this region are repaired only slightly slower than DSBs induced within the EC (Figure 3B). This result is most likely based on the mixed energy and LET of the ions within this region. The fraction of low-energy ions is small, and this is mirrored in the repair capacity. Similar results were obtained with one-field SOBP–carbon-ion irradiation (50 keV/μm dose-averaged LET) of non-synchronized hamster cells (47). Nonetheless, although the DSBs induced in the SOBP are repaired the RBE of therapy-relevant carbon-ion irradiation is increased [see above and Ref. (48)]. This leads to the assumption that misrepair plays a non-negligible role in the increased RBE. The proximity of the DSBs within clusters may enhance the probability of misrejoining. In addition, the pathway choice has an important impact on the accuracy of the DNA repair, and hence, will be discussed in greater detail. Figure 4 summarizes known and proposed repair activities at complex, ion-induced DSBs.

### Repair Pathways of Complex DSBs

The observation that DSBs induced in G0/G1-phase human fibroblasts within the SOBP are repaired slightly slower than DSBs induced within the EC (Figure 3B) suggests that DNA-repair pathways involving different types of end processing might be used. This is supported by earlier findings showing that with increasing LET an increasing number of resected DSBs is found. This occurs independent of the cell cycle phase (32, 49, 50) and is important for DSB repair (32). Notably, DSB repair involving resected ends represents a potential source of erroneous repair (33). The observed resection is dependent on MRE11, CtIP, and EXO1 (32, 49, 50). In addition, break-end processing by Artemis may take also place as this nuclease was shown to be important for the survival upon ion irradiation (9, 51) and was suggested to be involved in the repair of α particle-induced DSBs (52).

Which pathways repair DSBs induced by therapy-relevant carbon ions is still under investigation. Based on data on X-ray and carbon-ion irradiated human G2-phase cells, it was proposed that classical non-homologous end joining (c-NHEJ) will make an initial attempt to repair the DSBs (37, 53). This hypothesis is supported by data on proliferating hamster cells irradiated with SOBP–carbon ions, which show that c-NHEJ is vital to repair the induced DSBs (47). The choice of this pathway is supported by the findings that the repair of high-LET iron-ion (150 keV/μm) and α-particle (130 ± 10 keV/μm)-induced DSBs require DNA-PKcs, an important component of c-NHEJ (54). In addition, recruitment of GFP-tagged Ku80, a further important component of c-NHEJ, to DSBs induced by single gold ions was observed in living murine cells (55). If due to DNA fragmentation generated by ion irradiation the Ku complex cannot form fast enough (56), c-NHEJ may fail to proceed quickly. Then, resection of ion-induced DSBs by MRE11, CtIP, and EXO1 and break-end processing by Artemis may occur (32, 49, 50, 57). It is conceivable that Artemis in its function as endonuclease trims the resected DSB ends – either by opening hairpins that form from single-stranded stretches or by trimming off single-stranded areas (58, 59) – to make the break ends available for the c-NHEJ repair machinery (45, 52). DSBs with single-stranded overhangs will be channeled into homology-mediated repair. In G2-phase cells, this might be single strand annealing (SSA), but it mainly represents homologous recombination (HR) as was shown upon irradiation with carbon or iron ions (37, 47, 60). The fate of resected DSBs in G1-phase cells is mainly unknown. They are not repaired by HR (32, 60). c-NHEJ factors are discussed to be involved in a repair option involving a Ku-dependent microhomology-mediated end joining (MMEJ) pathway in G1-phase cells (45, 61). However, c-NHEJ itself is considered to be unable to repair DSB break ends with long single-stranded overhangs (33). Ku- and LIG4-independent alternative (alt)-NHEJ represents a further repair choice for G1-phase DSBs with long single-stranded overhangs (62, 63), since it frequently involves CtIP- and MRE11-dependent break resection and microhomologies for ligation (64–69). Although it was described to operate only if Ku is absent (70, 71), it was proposed to operate in repair proficient cells if c-NHEJ fails locally (7). The choice of microhomology-mediated pathways is supported by the fact that ion-induced, rejoined DSBs are often characterized by deletions and flanking microhomologies (72). It should be noted that besides HR all repair pathways using processed break ends are inherently erroneous.

An increased use of error-prone repair and the close proximity of the breaks, which could facilitate the ligation of wrong break ends, represent likely reasons for the increased mutation and chromosome-aberration rate seen in cells treated with high-LET radiation (72). Considering our here presented data, we propose that misrepair and thus mutations and aberrations play a non-negligible role in the increased RBE for cell killing of therapy-relevant carbon radiation.

## Materials and Methods

### Cells, Cell Culture, and Survival Assay

Normal human foreskin fibroblasts AG1522 (Coriell Cell Repository, Camden, NJ, USA; passage 11–15) were cultured in EMEM with EBSS salts, 15% FCS, 2 mM l-glutamine, and 1% penicillin/streptomycin at 37°C, 5% CO_2_. To obtain confluent cultures enriched in G1 cells, 10^4^ cells/cm^2^ were seeded and used for experiments 10 days later. For the survival assays, the clonogenic survival was determined, as described earlier (14). For the survival data in Figure 1, cells were cultivated on polystyrene slides (35). For the repair kinetics and the associated survival data, cells were cultivated on glass cover slips (ø 30 mm or 24 mm × 24 mm).

### Irradiation

Cells were irradiated with X-rays (250 keV, 16 mA; X-ray tube IV320-13, Seifert, Germany) or carbon ions at the GSI Helmholtz Center for Heavy Ion Research (Darmstadt, Germany). Irradiations with low-energy carbon ions were performed at the UNILAC beam line (11.4 MeV/u primary energy, 9.9 MeV/u on target, LET 168 keV/μm) and with high-energy carbon ions at the heavy-ion synchrotron (SIS) using active energy variation and raster scanning (48). Since the selection of ions available is limited some data are from single experiments only. For the survival data in Figure 1, cells were irradiated in a medium filled polyacrylic tank (35) (Figure 1A). The one-field carbon-ion irradiation was done with a 4-cm SOBP in a water-equivalent depth of 6–10 cm (LET: 45 keV/μm at the proximal edge, 150 keV/μm at the distal edge). For the repair kinetics and corresponding survival data, cells were exposed at different positions within a medium filled polyacrylic tank (Figure 2A). Cells seeded on glass cover slips were positioned approximately 3, 16, and 29 cm from the beam entrance side. To simulate the two-field configuration typical for patient irradiation, the tank was first irradiated from one side and after turning it horizontally by 180°, it was irradiated from the other side with the same dose distribution. An SOBP with a width of 2.4 cm at a water-equivalent depth of 16 cm was applied. The dose in the SOBP was 2 Gy and the dose-averaged LET values were about 70 and 85 keV/μm in the center and at the edges of the SOBP, respectively. Samples for SOBP irradiation were placed in the middle of the SOPB to minimize the influence of variations in positioning. Samples in the EC region were irradiated at a depth of a few millimeter, corresponding to a dose-averaged LET of 13 keV/μm and a dose of 0.6 Gy.

### Model Calculations

Model calculations were performed using the LEM, as described by Elsässer et al. (35). The model allows predicting the effects of ion radiation based on the localized, microscopic energy deposition pattern of particle tracks in combination with the knowledge of the photon dose–response curve for the endpoint under consideration. The corresponding parameters of the photon cell survival curve were α = 0.54 Gy^−1^, β = 0.062 Gy^−2^, and *D*_t_ = 13.5 Gy, where *D*_t_ characterizes the transition from a curvilinear shape at low and intermediate doses to a linear shape at high doses [for details see, e.g., Ref. (73)]. The dose–response of AG1522D cells on glass cover slips was estimated by scaling the dose values by a factor of 1.3 according to the information given in Furre et al. (36).

### Immunostaining

For the γH2AX-foci analyses, DSBs were visualized by γH2AX immunostaining performed, as described in Meyer et al. (42). The global immunofluorescent γH2AX signal was analyzed by flow cytometry according to Tommasino et al. (43).

## Author Contributions

GTS designed research; WKW and JT performed research; NBA, WKW, MS, and JT analyzed data; and NBA, MD, MS, and GTS wrote the paper. All authors approved the work for publication.

## Conflict of Interest Statement

The authors declare that the research was conducted in the absence of any commercial or financial relationships that could be construed as a potential conflict of interest.
